# Massive rectal bleeding from a superior mesenteric artery pseudoaneurysm successfully managed by coil embolization in an HIV-positive patient with disseminated tuberculosis: a case report

**DOI:** 10.11604/pamj.2026.53.8.42369

**Published:** 2026-01-09

**Authors:** Mariem Ben Ticha, Hela Knani, Nadia Ben Lasfar, Manel Ben Selma, Maha Abid, Yasser Ben Cheikh, Amel Omezzine Letaief, Wissem Hachfi, Foued Bellazreg

**Affiliations:** 1 Department of Infectious Diseases, Faculty of Medicine of Sousse, Farhat Hached University Hospital, Sousse, Tunisia

**Keywords:** Intestinal tuberculosis, pseudoaneurysm, gastrointestinal bleeding, embolization, HIV, case report

## Abstract

Gastrointestinal bleeding is a rare manifestation of intestinal tuberculosis, with massive hemorrhage from a mesenteric artery pseudoaneurysm representing an exceptionally uncommon and life-threatening complication. As illustrated by the case of a 27-year-old male with newly diagnosed HIV (CD4 count 10 cells/mm^3^) who presented with a 3-month history of diarrhea, fever, and significant weight loss. This led to a confirmed diagnosis of disseminated tuberculosis. On day 5 of anti-tuberculosis treatment, the patient developed massive hematochezia, and abdominal computed tomography (CT)-angiography followed by diagnostic arteriography identified a pseudoaneurysm of the appendicular branch of the ileocolic artery, which was successfully embolized with coils. This resulted in immediate hemostasis and stabilization of the patient, allowing for the initiation of antiretroviral therapy one month later. This case demonstrated that angioembolization is a highly effective and minimally invasive therapeutic alternative to surgery for this critical condition, even in immunocompromised patients.

## Introduction

Intestinal tuberculosis can present complications such as obstruction, perforation, and fistula formation. Lower gastrointestinal hemorrhage is an uncommon but potentially fatal complication, typically resulting from oozing mucosal ulcers [1]. However, massive bleeding caused by the erosion of a vessel and the formation of a pseudoaneurysm is exceedingly rare. While surgical intervention has been the traditional treatment, transcatheter arterial embolization has emerged as a viable and effective alternative [2]. We report a case of disseminated tuberculosis in an HIV-positive patient who presented with massive rectal bleeding from a superior mesenteric artery branch pseudoaneurysm, which was successfully managed with angiographic embolization.

## Patient and observation

**Patient information:** a 27-year-old male patient newly diagnosed with Human Immunodeficiency Virus (HIV), and prior to this admission, he had received a provisional misdiagnosis of Crohn's disease for which he was treated with a one-week course of corticosteroid therapy, which proved entirely unsuccessful in alleviating his symptoms.

**Clinical findings:** the patient presented to the hospital with a chief complaint of a three-month history of persistent diarrhea, fever, and profound, unintentional weight loss of 20 kilograms. Upon initial clinical evaluation at admission, the patient was afebrile with a body temperature of 36.4°C; physical examination revealed significant abdominal tenderness, clinical signs of dehydration, and the presence of palpable lymph nodes in both the cervical and axillary regions. Laboratory investigations uncovered severe anemia with a hemoglobin level of 7.6 g/dL, hypoalbuminemia at 23 g/L, and hyponatremia of 124 mmol/L.

**Timeline of current episode:** on 9^th^ February 2023, the patient was referred to the infectious diseases department for a newly diagnosed HIV infection. Between 9^th^ and 15^th^ February 2023, lymph node biopsies were performed for histological and microbiological analyses. On the 16^th^ of February 2023, anti-tuberculosis therapy was initiated. On 21^st^ February 2023, massive hematochezia and arteriography revealed a pseudoaneurysm at the appendicular branch of the ileocolic artery. On the 22^nd^ of February 2023, endovascular embolization was performed. In March 2023, antiretroviral therapy was initiated. In June 2023, the patient had gained 8 kg.

**Diagnostic assessment:** confirmatory HIV testing returned a positive result with viral load of 210,000 copies/mL and a critically low CD4 count of 10 cells/mm^3^. Thoraco-abdomino-pelvic computed tomography (CT) scan revealed a constellation of findings highly suggestive of a disseminated infectious process, including pulmonary nodules with signs of excavation, circumferential thickening of the terminal ileum and the ileocecal junction, and the presence of multiple necrotic lymph nodes.

Endoscopic evaluation was pursued, with a colonoscopy demonstrating severe acute ileitis and an esophagogastroduodenoscopy (EGD) identifying concomitant mycotic esophagitis and congestive gastro-bulbitis. Histopathological and microbiological analyses of various tissue samples were critical for a definitive diagnosis: an ileal biopsy showed chronic active inflammation but notably no granulomas; a sputum sample was negative for acid-fast bacilli; a biopsy of a cervical lymph node revealed inflammatory granulation tissue with necrosis, which, when tested with the GeneXpert MTB/RIF assay, confirmed the presence of rifampicin-sensitive *Mycobacterium tuberculosis*.

**Diagnosis:** the final diagnosis of disseminated tuberculosis was made.

**Therapeutic intervention:** a standard four-drug anti-tuberculosis therapy regimen consisting of isoniazid, rifampicin, ethambutol, and pyrazinamide was initiated.

**Figure 1:**
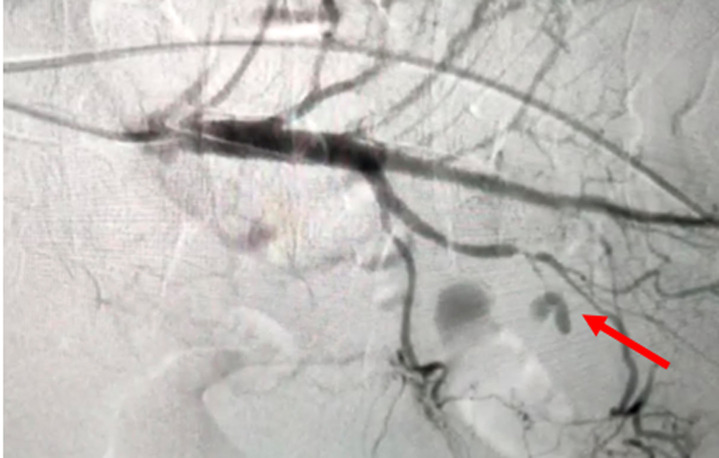
diagnostic arteriography image showing the pseudoaneurysm (false aneurysm) on the appendicular branch of the ileocolic artery

**Figure 2:**
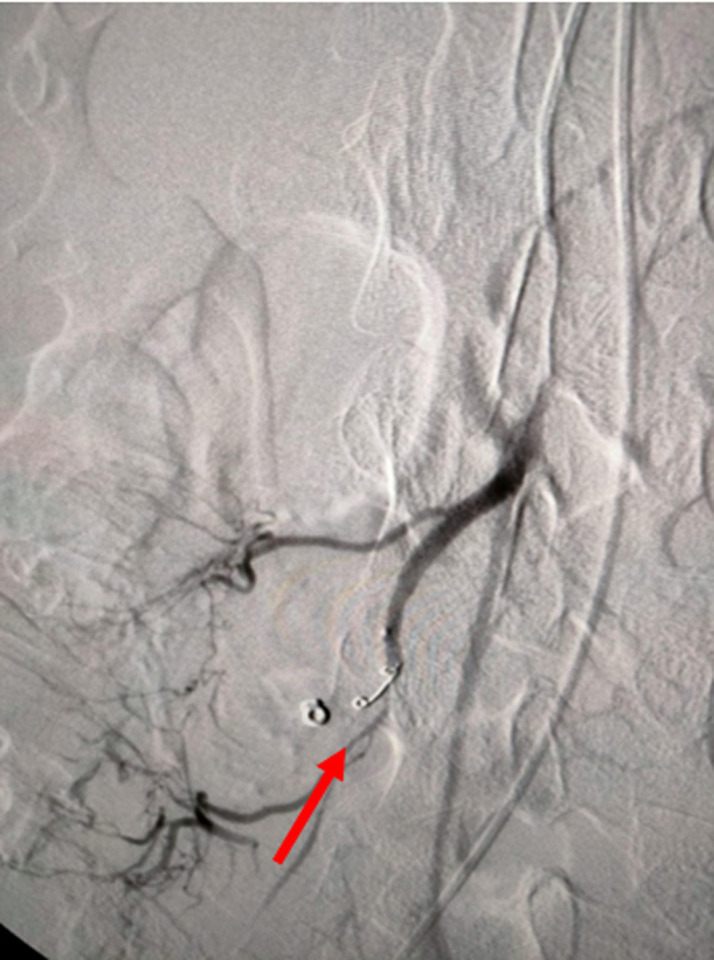
post-embolization control angiogram showing successful occlusion of the pseudoaneurysm with coils and maintained patency of the parent artery

**Follow-up and outcome of intervention:** on the fifth day of treatment, the patient experienced a severe complication in the form of massive hematochezia, prompting an urgent interventional response. A subsequent angiography procedure was performed, which identified the source of the hemorrhage as a pseudoaneurysm of the appendicular branch of the ileocolic artery, a branch of the superior mesenteric artery (Figure 1); this life-threatening lesion was then successfully treated via endovascular embolization using four Vortex 0.18 coils, each with a diameter of 3mm (Figure 2).

The patient's outcome was excellent. Post-embolization, there was no further rectal bleeding, and his hemodynamic status stabilized. Antiretroviral therapy for his HIV, specifically a regimen of tenofovir, lamivudine, and dolutegravir, was initiated one month after the bleeding event. After several months of follow-up, he achieved complete symptom resolution and a weight gain of 8 kilograms.

**Patient perspective:** “*Being diagnosed with disseminated tuberculosis and experiencing massive rectal bleeding was shocking, but after embolization and starting treatment, I became determined to adhere strictly to my medications and follow-up, hoping to fully recover*”.

**Informed consent:** written informed consent was obtained from the patient.

## Discussion

Abdominal tuberculosis represents a significant proportion of extrapulmonary tuberculosis cases, capable of involving the gastrointestinal tract, peritoneum, solid organs, and lymph nodes [3]. Gastrointestinal tuberculosis (GITB) is the most prevalent form, accounting for 43-65% of abdominal tuberculosis cases, with a predilection for the ileocecal region (44-84% of cases) [3]. Immunocompromised states, particularly HIV infection with low CD4 counts, are well-established risk factors for extrapulmonary and abdominal tuberculosis [3]. The clinical presentation of GITB is often non-specific, encompassing abdominal pain, distension, altered bowel habits, and nausea, with severe complications including perforation and peritonitis [3-5].

Gastrointestinal bleeding is an uncommon but serious complication of GITB, occurring in only 1-5% of cases [5]. While typically caused by bleeding from deep ulcers secondary to obliterative endarteritis [1,6], massive hemorrhage can result from the rupture of a mycotic pseudoaneurysm. These pseudoaneurysms are rare sequelae of tuberculosis, arising from the spread of infection to the arterial wall via direct extension, hematogenous, or lymphangitic routes, leading to mural necrosis. Cases of intra-abdominal tuberculous pseudoaneurysms affecting vessels such as the celiac, aortic, gastroduodenal, and superior mesenteric arteries have been reported in both adult and pediatric populations [1,7-9].

The diagnostic challenge of GITB lies in its atypical presentation, necessitating a multifaceted approach involving microbiological, radiological, and endoscopic investigations. Common laboratory findings include anemia (22-90% of cases) and hypoalbuminemia (approximately 44%) [3]. Cross-sectional imaging with CT is valuable for identifying mural and extramural changes, with positive findings in up to 88% of patients; however, these features are often non-specific and can mimic other inflammatory conditions such as Crohn's disease [5], as was initially suspected in the present case. Endoscopy with biopsy is crucial, yet histopathological confirmation can be elusive, with granulomas identified in only 62-71% of cases [10].

The primary treatment for GITB is standard anti-tuberculosis chemotherapy. However, a subset of patients with complications, including obstruction (66%), perforation (29%), aneurysm, or bleeding (6%), may require surgical intervention [3]. Surgical mortality rates are significant, ranging from 14% to 20%, and can exceed 50% in emergent settings [7]. In such high-risk scenarios, transcatheter arterial embolization has emerged as a successful minimally invasive alternative for managing visceral artery aneurysms, including those of the superior mesenteric artery [5,7]. In the case of our patient, this approach averted the need for high-risk surgery; urgent angiography localized the bleeding source, and subsequent coil embolization successfully achieved hemostasis.

## Conclusion

Intestinal tuberculosis can rarely present with massive gastrointestinal bleeding, particularly in cases involving rupture of a mesenteric artery pseudoaneurysm. Although uncommon, this diagnosis should be considered in any patient presenting with acute gastrointestinal hemorrhage in the context of known or suspected tuberculosis. Angioembolization represents an effective and less invasive therapeutic alternative to surgery, enabling rapid hemorrhage control and improved outcomes. This case highlights the importance of prompt, multidisciplinary management in such life-threatening scenarios.
